# NADPH Oxidase-4 Driven Cardiac Macrophage Polarization Protects Against Myocardial Infarction–Induced Remodeling

**DOI:** 10.1016/j.jacbts.2017.06.006

**Published:** 2017-12-25

**Authors:** Heloise Mongue-Din, Ashish S. Patel, Yee H. Looi, David J. Grieve, Narayana Anilkumar, Alexander Sirker, Xuebin Dong, Alison C. Brewer, Min Zhang, Alberto Smith, Ajay M. Shah

**Affiliations:** a King's College London British Heart Foundation Centre, Cardiovascular Division, James Black Centre, London, United Kingdom; b King's College London British Heart Foundation Centre, Cardiovascular Division, Academic Department of Vascular Surgery, St. Thomas' Hospital, London, United Kingdom

**Keywords:** cardiac, infarction, ischemia, macrophage, NADPH oxidase, remodeling, IL, interleukin, I/R, ischemia/reperfusion, LAD, left anterior descending coronary artery, LV, left ventricular, MI, myocardial infarction, MMP, matrix metalloproteinase, mRNA, messenger ribonucleic acid, NF-κB, nuclear factor–κB, Nox, NADPH oxidase, TG, transgenic, WT, wild-type

## Abstract

The reactive oxygen species–generating enzyme NADPH oxidase 4 (Nox4) is up-regulated in the heart after myocardial infarction (MI). Mice with cardiomyocyte-targeted Nox4 overexpression (TG) displayed increased macrophages in the heart at baseline, with skewing toward an M2 phenotype compared with wild-type controls (WT). After MI, TG mice had a higher proportion of M2 macrophages along with higher survival, decreased cardiac remodeling, and better contractile function than wild-type mice. The post-MI increase in cardiac matrix metalloproteinase–2 activity was substantially blunted in TG mice. These results indicate that cardiomyocyte Nox4 modulates macrophage polarization toward an M2 phenotype, resulting in improved post-MI survival and remodeling, likely through the attenuation of cardiac matrix metalloproteinase–2 activity.

## Highlights

• The reactive oxygen species–generating enzyme NADPH oxidase 4 (Nox4) is up-regulated in the heart after myocardial infarction.
• Mice with cardiomyocyte-targeted overexpression of Nox4 display an increase in macrophages in the heart, with skewing of polarization toward an M2 phenotype.
• After myocardial infarction, Nox4-induced skewing of macrophage polarization toward an M2 phenotype is accompanied by a higher survival, decreased cardiac remodeling, and improved contractile function.
• The protective effects of cardiomyocyte Nox4 may, in part, involve the attenuation of cardiac matrix metalloproteinase–2 activity.

## Visual Abstract

**Figure:**
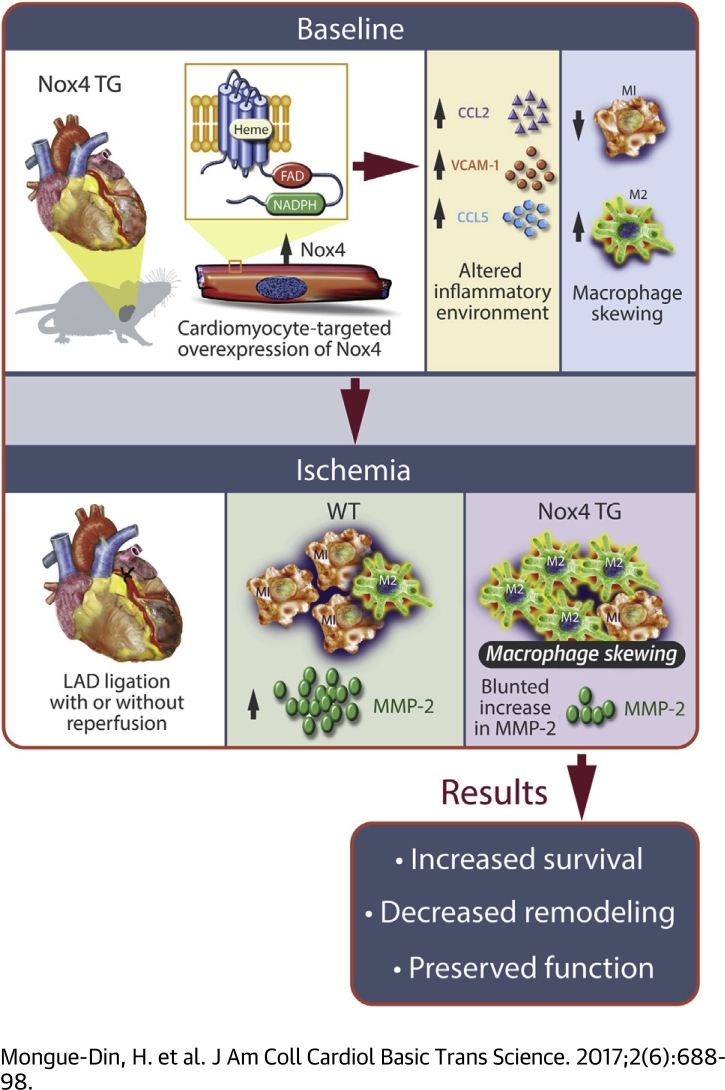

Myocardial infarction (MI) is a major cause of heart failure. Moderate to large MI causes chronic left ventricular (LV) remodeling that eventually results in dilatation, interstitial fibrosis, impaired contraction, and life-threatening arrhythmia. The initial response to MI significantly influences post-MI remodeling. Immediately after MI, there is an acute inflammatory phase involving significant tissue infiltration by neutrophils, monocytes/macrophages, and lymphocytes (1). These inflammatory cells orchestrate initial infarct repair through the clearance of dead cardiomyocytes and debris. This scenario is followed by laying down of scar tissue and scar maturation, along with gradual loss of inflammatory cells. Successful healing of the infarct and the transition to a mature scar that favors preservation of overall LV function is greatly influenced by a harmonious balance between these phases and the pattern of inflammatory changes that occur in the heart.

Numerous studies in experimental MI have established that the pattern of macrophage infiltration is especially important 1, 2, 3. Monocytic cell lineage is heterogeneous and characterized by a high plasticity in response to environmental signals (4). Healing and remodeling after acute MI involve the successive recruitment of different macrophage subsets with complementary functions, commonly termed classically activated (“M1”) and alternatively activated (“M2”) macrophages, respectively 2, 5. In the first few days after MI, there is a peak in M1 macrophages, cells that have a high phagocytic activity and produce abundant pro-inflammatory cytokines 2, 3. The number of M2 macrophages in the infarct peaks later, and these more heterogeneous cells are believed to tune an anti-inflammatory, reparative response.

Reactive oxygen species (ROS) signaling is involved in the development of many components of the failing heart phenotype, such as cardiomyocyte hypertrophy, cell death, extracellular matrix remodeling, and chamber dilatation (6). NADPH oxidase (Nox) family enzymes are known to be important modulators of redox signaling in heart failure. Unlike ROS sources such as mitochondria or xanthine oxidase, ROS production appears to be the primary function of Nox proteins (7). Of the 5 known Nox family proteins, Nox2 and Nox4 are expressed in the murine heart and have distinct roles in different cardiac pathologies. Nox2 activation contributes to adverse remodeling post-MI, where it promotes cardiomyocyte death, hypertrophy, and extracellular matrix remodeling, as well as being involved in cardiac rupture (6). Nox4 differs from Nox2 in that it is constitutively active, is regulated mainly at the level of abundance, and generates predominantly H_2_O_2_ rather than superoxide as its initial product 7, 8. Previous research also showed that Nox4 differs from most other ROS sources by exerting beneficial effects in the heart during chronic pressure overload or starvation, where its expression level increases and enhances specific redox-sensitive signaling 9, 10. Nox4 has been linked to anti-inflammatory effects in the vasculature, where it ameliorates inflammation, remodeling, and atherosclerosis 11, 12. Myocardial Nox4 levels are increased after acute MI 9, 10, but its possible role in modulating inflammation in the heart has not been studied.

The present study investigated the effects of cardiomyocyte Nox4 on inflammation and remodeling after acute MI. Using a transgenic mouse model with cardiomyocyte-targeted Nox4 overexpression in a pathophysiologically relevant range, we found that Nox4 modifies macrophage polarization such that ventricular remodeling, contractile function, and survival are improved after acute MI.

## Materials and Methods

### Experimental MI

Animal procedures were conducted in accordance with the Guidance on the Operation of the Animals (Scientific Procedures) Act, 1986 (UK Home Office). Mice with cardiomyocyte-targeted overexpression of Nox4 (TG) have been described previously (9) and were compared with matched wild-type (WT) littermates. Female TG and WT animals approximately 12 weeks of age and on a C57Bl/6 background underwent left anterior descending coronary artery (LAD) ligation under 2% isoflurane/98% oxygen anesthesia (13). Animals underwent permanent ligation or 30 min ligation followed by reperfusion (ischemia/reperfusion [I/R]). Sham procedures were identical except for ligation.

### In vivo analyses

Echocardiography was performed under 1.5% isoflurane anesthesia (9). LV pressure was measured under 2% isoflurane anesthesia via carotid artery cannulation using a 1.4-F micromanometer-pressure catheter (SPR-839, Millar Instruments, Houston, Texas).

### Histology

Hearts arrested in diastole were used for histologic assessments (9). Paraffin sections were stained with Picrosirius red to assess collagen content and rhodamine-conjugated wheat germ agglutinin to quantify cardiomyocyte cross-sectional areas. Cryosections were stained with an anti–F4/80 antibody (ab16911, Abcam, Cambridge, Massachusetts) to identify macrophages. Imaging was done on an IX81 microscope (Olympus UK, Southend-on-Sea, United Kingdom). Image J software (National Institutes of Health, Bethesda, Maryland) was used for quantification. Six representative fields at ×20 magnification (3 per section; 2 sections per heart) were used per animal.

To quantify initial infarct size after permanent coronary ligation, Evans blue dye (1%) was perfused retrogradely into the aorta 24 h after MI. Hearts were sliced into 5 serial transverse sections and incubated in 1% triphenyltetrazolium chloride to identify infarcted myocardium (13). Infarct area as a percentage of total LV area was quantified by computerized planimetry. Infarct size at 4 weeks’ post-MI was determined as the total infarct circumference indexed to total LV circumference (14).

### Real-time reverse transcription–polymerase chain reaction

We used the comparative cycle threshold method with SybrGreen, using β-actin for normalization. Primer sequences (forward, reverse) were: EMR1: TGAATGGCTCCATTTGTGAA, GGCCCTCCTCCACTAGATTC; CCR2: ACCTGTAAATGCCATGCAA, TGTCTTCCATTTCCTTTGAT; CCL2: CATCCACGTGTTGGCTCA, GATCATCTTGCTGGTGAATGAGT; CCL5: TGCAGAGGACTCTGAGACAGC, GAGTGGTGTCCGAGCCATA; VCAM-1: TCTTACCTGTGCGCTGTGAC, ACTGGATCTTCAGGGAATGAGT; TNF-α: TCCCAGGTTCTCTTCAAGGGA, GGTGAGGAGCACGTAGTCGG; IL-6: GCTACCAAACTGGATATAATCAGGA, CCAGGTAGCTATGGTACTCCAGAA; IL-10: CATGGGTCTTGGGAAGAGAA, AACTGGCCACAGTTTTCAGG; MRC1: CATGAGGCTTCTCCTGCTTC, CAAGTTGCCGTCTGAACTGA; CX3CR1: AAGTTCCCTTCCCATCTGCT, CAAAATTCTCTAGATCCAGTTCAGG; MMP2: GGGCTTCTGTCCTGACCA, AAGTTCTTGGTGTAGGTGTAGATCG; SOD1: GGACCTCATTTTAATCCTCACTCTAAG, GGTCTCCAACATGCCTCTCTTC; SOD2: CACACATTAACGCGCAGATCA, GGTGGCGTTGAGATTGTTCA; SOD3: ACACCTTAGTTAACCCAGAAATCTTTTC, GGGATGGATCTAGAGCATTAAGGA; Nox2: TTGAAGGGAGGAGGCATGAA, CAGCTTACAGACTGGAACTAGAAGTGTT; and β-actin: CGTGAAAAGATGACCCAGATCA, TGGTACGACCAGAGGCATACAG.

### Flow cytometry

Macrophage content and phenotype were analyzed by using flow cytometry. Hearts were perfused in vivo with saline to flush out blood. The whole left ventricle or infarct and remote regions were digested by incubation in collagenase type IV, DNase, and hyaluronidase for 30 min at 37°C, then filtered through a 70-μm mesh. Cell suspensions were washed and blocked with anti–CD16/CD32 before staining. Macrophages were identified as CD45^+^, lineage negative (CD19^–^, CD3^–^, NK1.1^–^, Ly6G^–^), CD11b^+^F4/80^+^ cells and quantified for Ly6C and MRC1 (CD206) expression by using a FACS Canto II flow cytometer (BD Biosciences, San Jose, California). For analysis of blood, monocytes were identified as CD45^+^, lineage negative (CD19^–^, CD3^–^, NK1.1^–^, Ly6G^–^), CD11b^+^ and CD115^+^ cells, and analyzed for Ly6C and MRC1 expression. Data were analyzed by using FlowJo software (Tree Star Inc., Ashland, Oregon).

### Zymography

Novex Pre-Cast Zymogram gels (Thermo Fisher Scientific, Waltham, Massachusetts) were used to detect and characterize matrix metalloproteinase (MMP)-2 three days after MI, using whole heart tissue. Proteins were extracted in lysis buffer and samples were denatured in sodium dodecyl sulfate–containing buffer under nonreducing conditions without heating. After electrophoresis, the enzyme was renatured by incubating the gel in Zymogram Renaturing Buffer containing a nonionic detergent. Gels were equilibrated in Zymogram Developing Buffer, then stained with Coomassie Brilliant Blue R-250 (Bio-Rad Laboratories, Hercules, California). Quantification was performed by using Image J software.

### Statistical analysis

Data are reported as mean ± SEM. Comparisons were undertaken on GraphPad Prism 5.00 (GraphPad Software, Inc., La Jolla, California) by using the Student *t* test or two-way analysis of variance followed by Bonferroni’s post-test, as appropriate. Kaplan-Meier survival analysis by the log-rank test was performed over a 4-week period after MI. Values of p < 0.05 were considered significant.

## Results

### TG mice are protected against cardiac dysfunction after I/R

**Figure 1:**
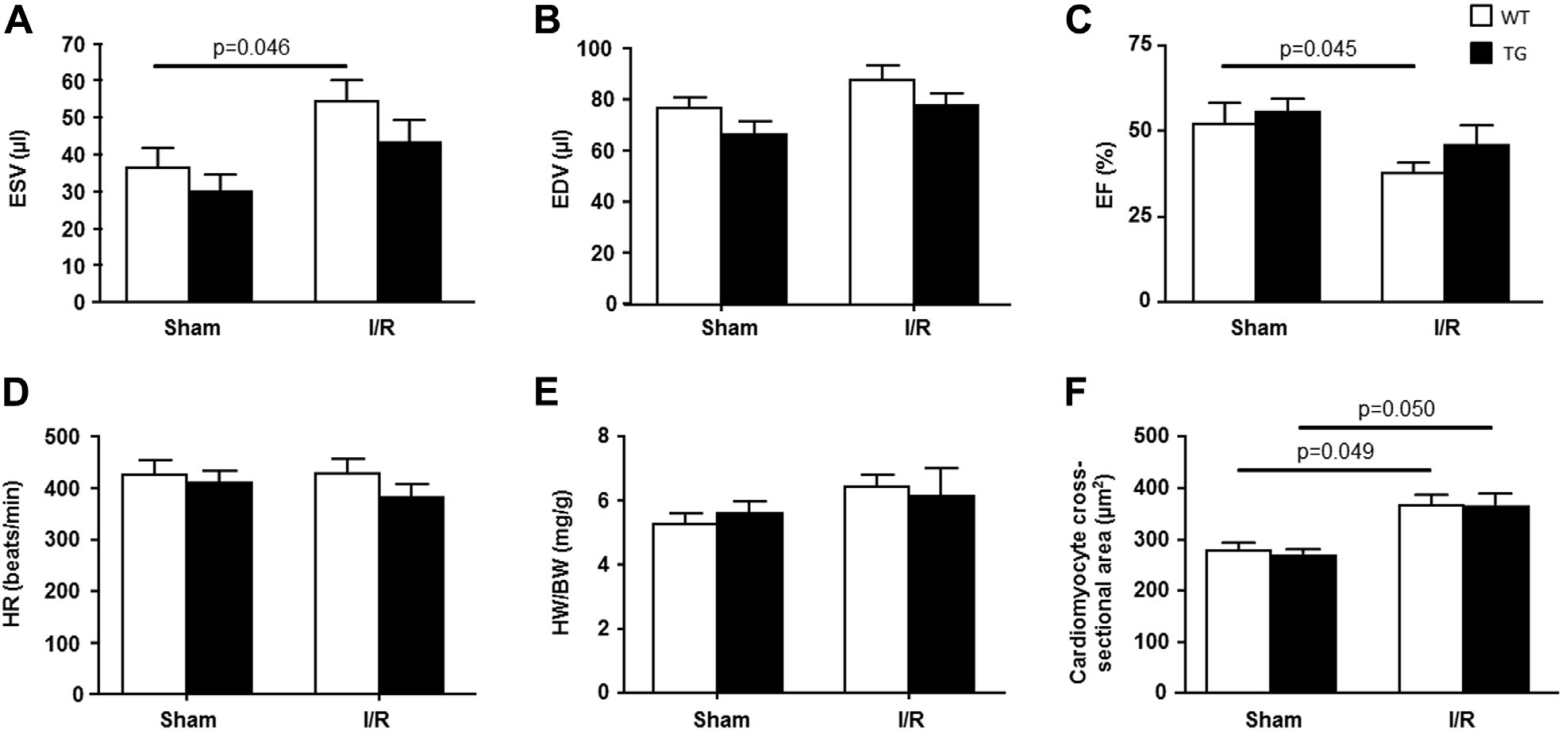
Cardiac Structure and Function in Nox4 TG Mice and Controls 1 Week After Reperfused MI **(A to D)** Changes in left ventricular end-systolic volume (ESV), end-diastolic volume (EDV), ejection fraction (EF), and heart rate (HR) assessed by echocardiography. **(E)** Heart weight/body weight ratio (HW/BW). **(F)** Cardiomyocyte cross-sectional area quantified in left ventricular sections. n = 5 to 6 per group. I/R = ischemia/reperfusion; MI = myocardial infarction; Nox4 = NADPH oxidase 4; TG = transgenic; WT = wild-type.

Overexpression of cardiomyocyte Nox4 in this model was accompanied by a <2-fold increase in H_2_O_2_ levels but no change in protein levels of Nox2 or in superoxide levels (9). There was also no difference in the expression of superoxide dismutases 1 through 3 between TG and WT hearts (Supplemental Figure 1). We first compared the functional response in TG and WT mice 1 week after I/R, a setting in which there is significant inflammatory infiltration in the heart. TG and WT sham groups exhibited similar cardiac function (Figures 1A to 1D), consistent with previous data that the modest increase in ROS levels in this model has no effect on baseline function. After I/R, WT animals showed a significant increase in LV end-systolic volume and a decrease in ejection fraction (Figures 1A to 1C) with no changes in heart rate (Figure 1D). In contrast, TG showed a preservation of LV end-systolic volume and ejection fraction after I/R. The heart/body weight ratio and cardiomyocyte cross-sectional area increased after I/R in both groups, but the changes were similar among groups (Figures 1E and 1F).

### Nox4 up-regulation modulates the basal inflammatory profile

**Figure 2:**
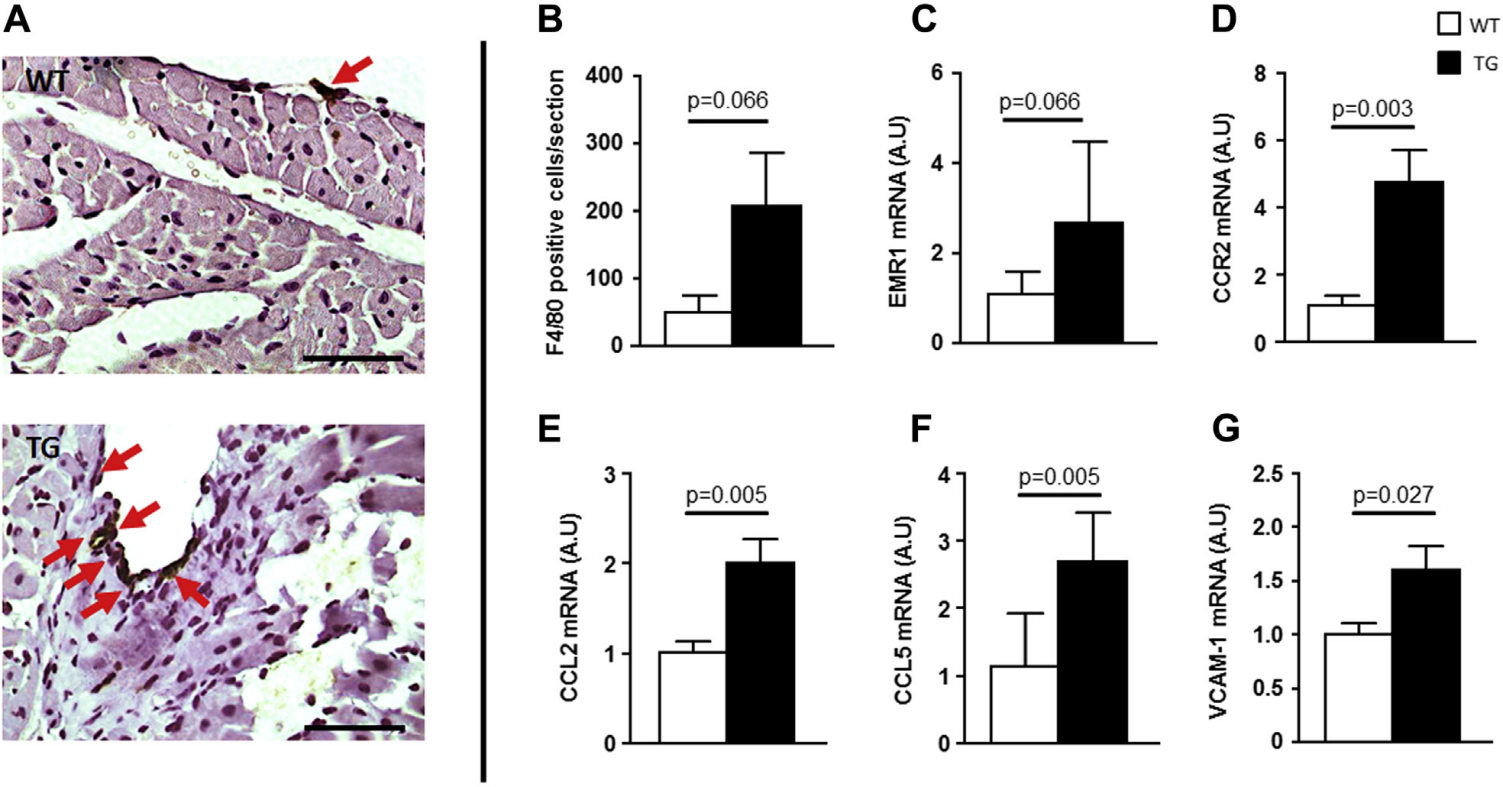
Nox4 Overexpression Alters Basal Inflammatory Profile in the Heart **(A and B)** Staining and quantification of macrophages (F4/80+ cells, **arrows**) in cardiac sections from TG and WT mice. Scale bar: 100 μm. **(C to G)** Messenger ribonucleic acid (mRNA) expression levels of macrophage-specific markers and chemo-attractant molecules in TG and WT heart. n = 5 to 6 per group. EMR1 = EGF-like module-containing mucin-like hormone receptor-like 1; CCR2 = C-C chemokine receptor type 2; CCL2 = C-C motif chemokine ligand 2; CCL5 = C-C motif chemokine ligand 5; VCAM = vascular cell adhesion molecule; other abbreviations as in Figure 1.

Inflammatory infiltration was first assessed by immunostaining myocardial sections with a macrophage marker (F4/80). Unexpectedly, there were differences between TG and WT mice even in the absence of MI, observed both in sham-operated animals and naive (unoperated) mice. The overall number of F4/80^+^ cells at baseline tended to be higher in naive TG mice compared with WT mice, and TG mice showed areas of macrophage accumulation that were not observed in WT mice (Figures 2A and 2B). Messenger ribonucleic acid (mRNA) levels of EMR1, the gene encoding F4/80, also tended to be higher in TG mice compared with WT mice (Figure 2C). More detailed analyses of inflammatory markers revealed significant increases in mRNA levels of the monocyte chemoattractants CCL2 and CCL5, the CCL2 receptor CCR2, and the vascular cell adhesion molecule–1 in TG mice compared with WT mice (Figures 2D to 2G). These results suggest that an increase in myocardial Nox4 levels alters the tissue inflammatory environment at baseline.

### Nox4 modulates cardiac macrophage polarization after I/R

We next assessed the response to I/R. To examine the quantity and nature of macrophage accumulation with higher fidelity, flow cytometry of whole cardiac tissue was used 3 days after I/R and sham ligation (Supplemental Figure 2). Lineage negative cells within the CD11b^+^ subset of the CD45^+^ population that expressed F4/80^+^ were identified as macrophages. The expression of Ly6C and MRC1 was used to distinguish between M1 (either Ly6C^high^ or MRC1^–^) and M2 (either Ly6C^low^ or MRC1^+^) phenotypes.

**Figure 3:**
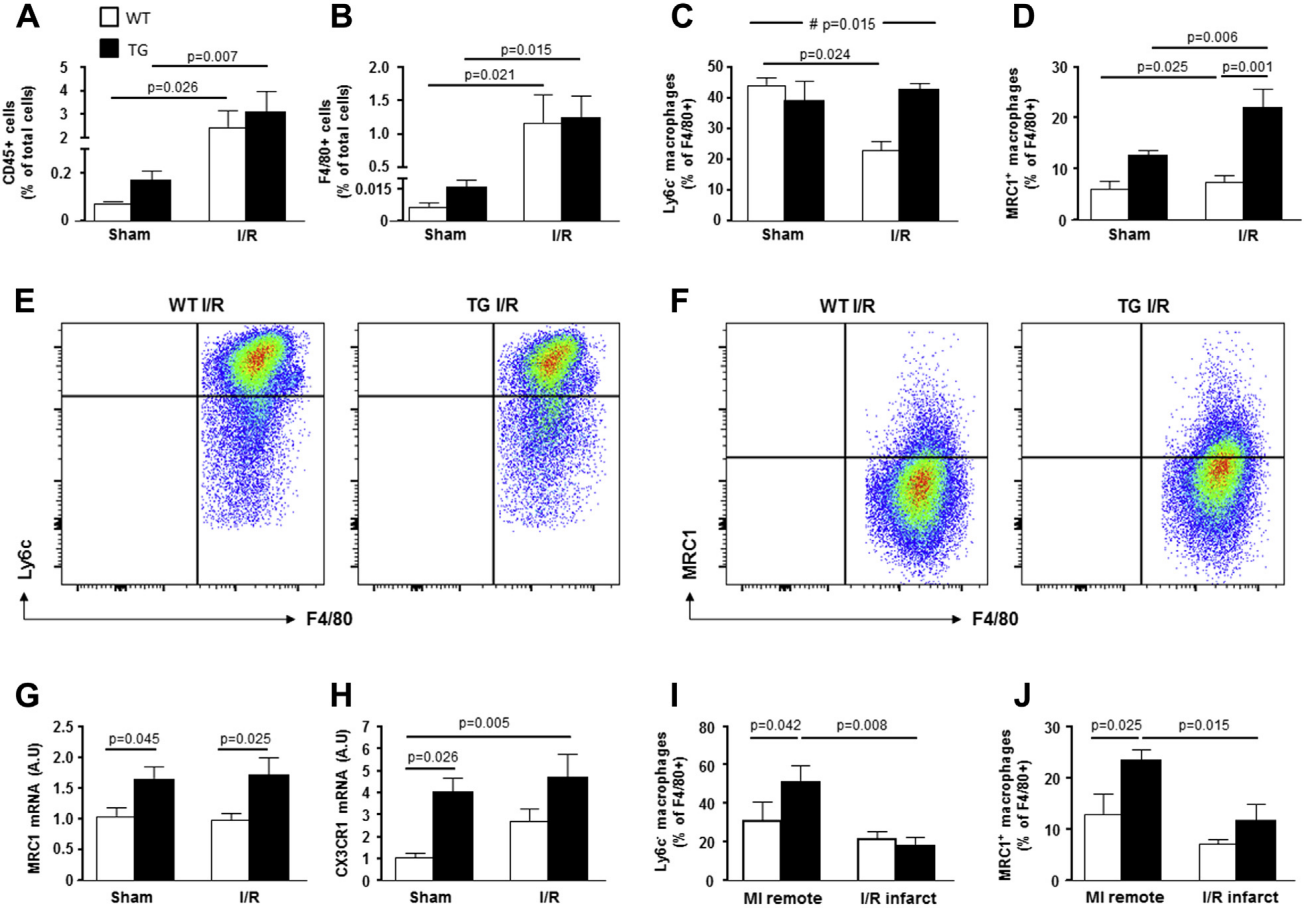
Nox4 Modulates Cardiac Macrophage Polarization After I/R **(A to D)** Flow cytometric quantification of total leukocytes, macrophages, and macrophage subsets according to Ly6c or MRC1 expression in the TG and WT left ventricle, 4 days’ post-MI. **(E and F)** Representative pseudocolor plots for macrophages based on Ly6c or MRC1 expression. **(G and H)** mRNA levels of the M2 markers MRC1 and CX3CR1 in whole left ventricle. **(I and J)** Ly6c- and MRC1+ macrophages as a proportion of total macrophages in the remote and ischemic areas of the left ventricle. n = 5 to 6 per group. #Significant interaction according to 2-way analysis of variance. Abbreviations as in Figures 1 and 2.

The total number of leukocytes (CD45^+^) was significantly and similarly increased after I/R in both TG and WT hearts (Figure 3A). Although CD11b^+^/F4/80^+^ macrophages as a percentage of total cells tended to be higher in TG than WT sham (p = 0.057), they increased to a similar level in both groups after I/R (Figure 3B). The proportion of Ly6C^low^ macrophages relative to total macrophages was similar in the 2 sham groups but was substantially higher in TG than WT after I/R (Figure 3C). The proportion of MRC1^+^ macrophages was significantly higher in TG compared with WT sham, and this difference was more pronounced after I/R (Figure 3D). Representative pseudocolor flow cytometric plots of the macrophage subsets are shown in Figures 3E and 3F. Consistent with these data, mRNA levels of MRC1 and CX3CR1 (markers of M2 macrophages) were significantly higher in TG myocardium than WT myocardium, both before and after I/R (Figures 3G and 3H). The proportion of circulating monocytes expressing Ly6C^low^ and MRC1^+^ were not significantly different between TG and WT groups either before or after I/R (Supplemental Figure 3). There was a significant increase in mRNA levels of interleukin (IL)-6 after I/R in WT hearts, which was markedly blunted in TG hearts, whereas levels of IL-10 and tumor necrosis factor–α were not significantly different between groups (Supplemental Figure 4).

We also separately analyzed changes in the infarct region and remote myocardium. Total CD45^+^ cells and CD11b^+^/F4/80^+^ macrophages were significantly higher in the infarct than in the remote region after I/R (Supplemental Figures 5A to 5C). TG remote myocardium had a significantly higher proportion of Ly6C^low^ cells and MRC1^+^ cells than WT remote myocardium, whereas the proportions were similar in the infarct region (Figures 3I and 3J, Supplemental Figures 5D and 5E). Thus, the TG remote myocardium had a predominance of Ly6C^low^ macrophages, whereas the WT remote myocardium had predominantly Ly6C^high^ cells.

Taken together, these results indicate that an elevation in cardiomyocyte Nox4 levels is accompanied by a modest increase in M2 macrophages in the myocardium, and that after I/R, there is a marked skewing toward an M2 phenotype (particularly in the noninfarct region).

### Effect of Nox4 on macrophage polarization after permanent LAD ligation

**Figure 4:**
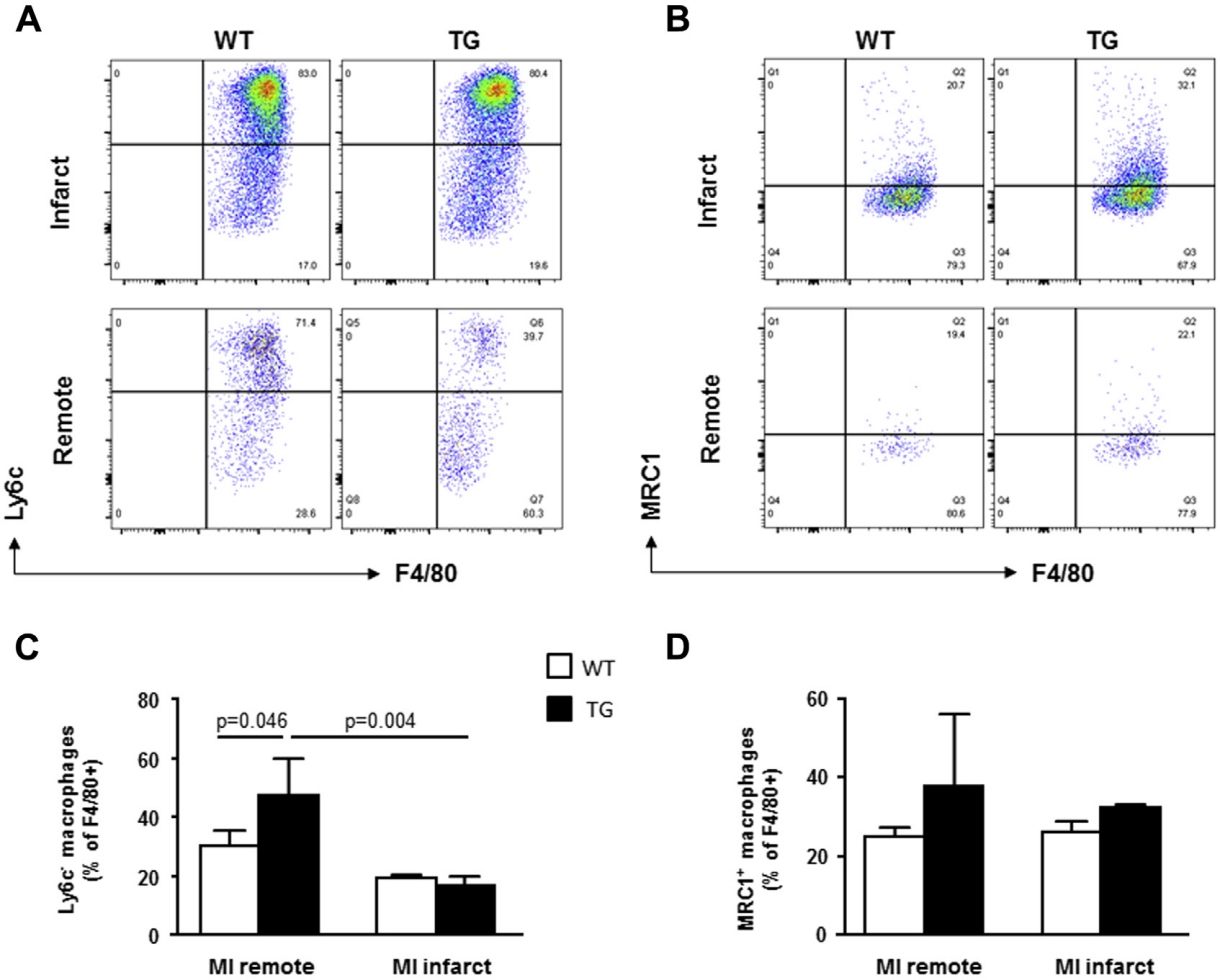
Nox4 Modulates Cardiac Macrophage Polarization After Permanent LAD Ligation Cardiomyocyte Nox4 drives polarization in a region-specific manner after permanent left anterior descending coronary artery (LAD) ligation. **(A and B)** Representative pseudocolor plots for macrophages based on Ly6c or MRC1 expression. Nox4 drives an inversion of the Ly6c expression pattern in the remote myocardium of TG. **(C and D)** Ly6c^-^ and MRC1^+^ macrophage proportions in the infarct and remote area. n = 5 to 6 per group. Abbreviations as in Figure 1.

We investigated whether similar changes in macrophage polarization occur after nonreperfused infarction. Three days after permanent LAD ligation, there was a profound leukocyte infiltration in the infarct, with a substantial number of macrophages (data not shown). Similar to the I/R model, a significant change in the Ly6C^low^/Ly6C^high^ balance was noted in the remote regions of TG compared with WT after permanent ligation (Figures 4A and 4C). The proportion of MRC1^+^ macrophages also tended to be higher in the remote area of TG compared with WT (Figures 4B and 4D). The proportion of Ly6C^low^ or MRC1^+^ macrophages in the infarct region were similar in WT and TG. The proportion of circulating Ly6C^low^ or MRC1^+^ blood monocytes were also similar in WT and TG groups (Supplemental Figure 3).

### Effect of Nox4 on survival and adverse remodeling after permanent LAD ligation

**Figure 5:**
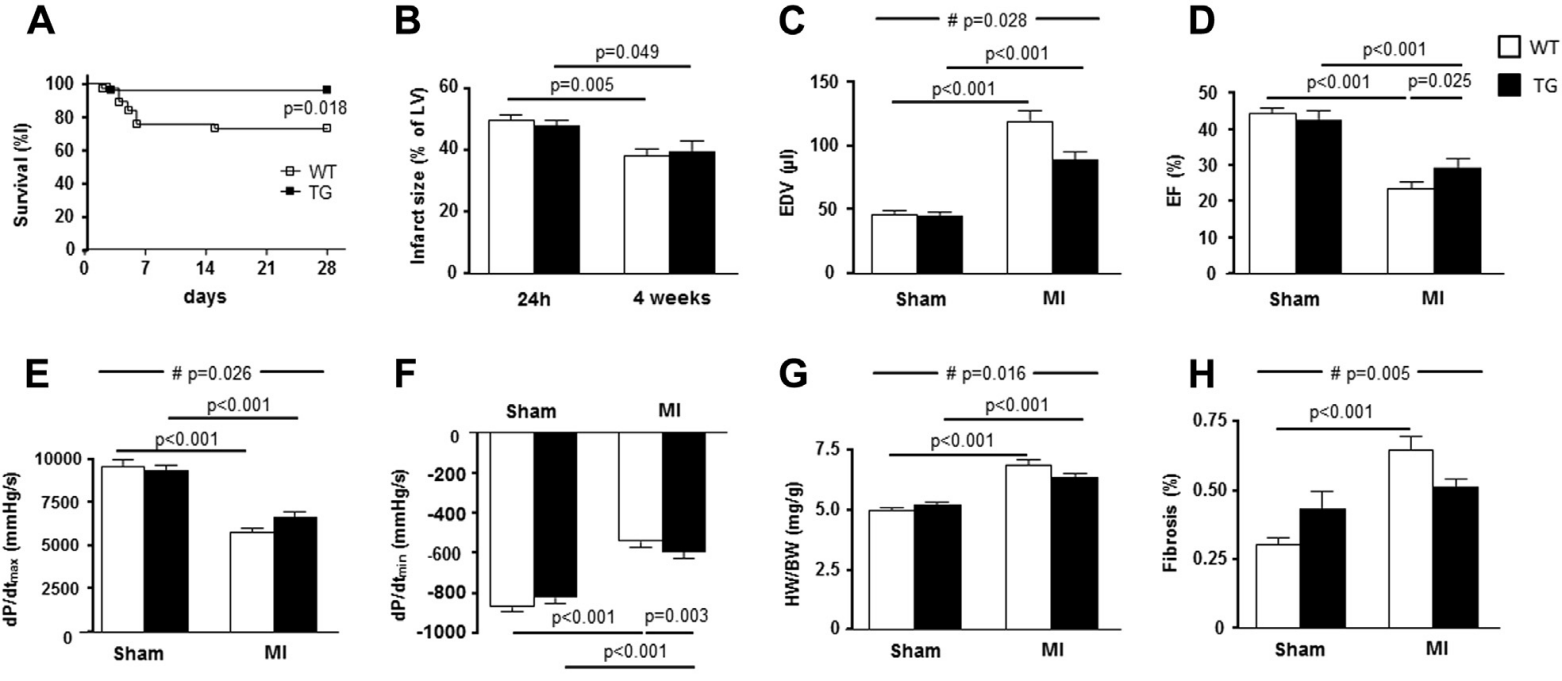
Cardiomyocyte-Targeted Nox4 Overexpression Improves Post-MI Survival and Protects Against Adverse Remodeling **(A)** Kaplan-Meier survival curves after MI in TG and WT (mouse numbers at 0, 7, 14, 21, and 28 days after MI: TG, 27, 26, 26, 26, and 26; WT, 37, 28, 28, 27, and 27). **(B)** Infarct size 24 h and 4 weeks after MI. n = 6 per group. **(C and D)** Echocardiographically quantified left ventricular EDV and EF after MI. n ≥ 15 per group. **(E and F)** Invasive cardiac contractile function assessed by maximum derivative of left ventricular pressure (dP/dt_max_) and minimum derivative of left ventricular pressure (dP/dt_min_). n = 8 per group. **(G)** Postmortem HW/BW. **(H)** Interstitial fibrosis in remote myocardium of TG and WT hearts after MI. n = 6 per group. #Significant interaction according to 2-way analysis of variance. LV = left ventricle; other abbreviations as in Figure 1.

TG had a significantly higher survival rate than WT after permanent LAD ligation (Figure 5A), which was related to a lower rate of cardiac rupture in the first few days after MI. Infarct size quantified 24 h after MI was similar in TG and WT (Figure 5B). Animals that survived beyond the first week were followed up for 28 days to assess post-MI remodeling. TG mice developed less LV dilatation (lower LV end-diastolic volume) and better preserved ejection fraction than WT after MI (Figures 5C and 5D). Infarct area at 4 weeks’ post-MI was similar in the 2 groups. Invasive hemodynamics revealed that TG had better LV systolic and diastolic function than WT, as assessed by maximum derivative of LV pressure and minimum derivative of LV pressure, respectively (Figures 5E and 5F). TG had a lower heart/body weight ratio than WT 4 weeks after MI (Figure 5G) and showed less interstitial fibrosis in the remote myocardium (Figure 5H, Supplemental Figure 6). Nox2 expression was similar among groups (Supplemental Figure 7). Taken together, these data indicate that TG are protected against early death and late adverse remodeling after MI.

### Cardiomyocyte Nox4 alters myocardial MMP activation

**Figure 6:**
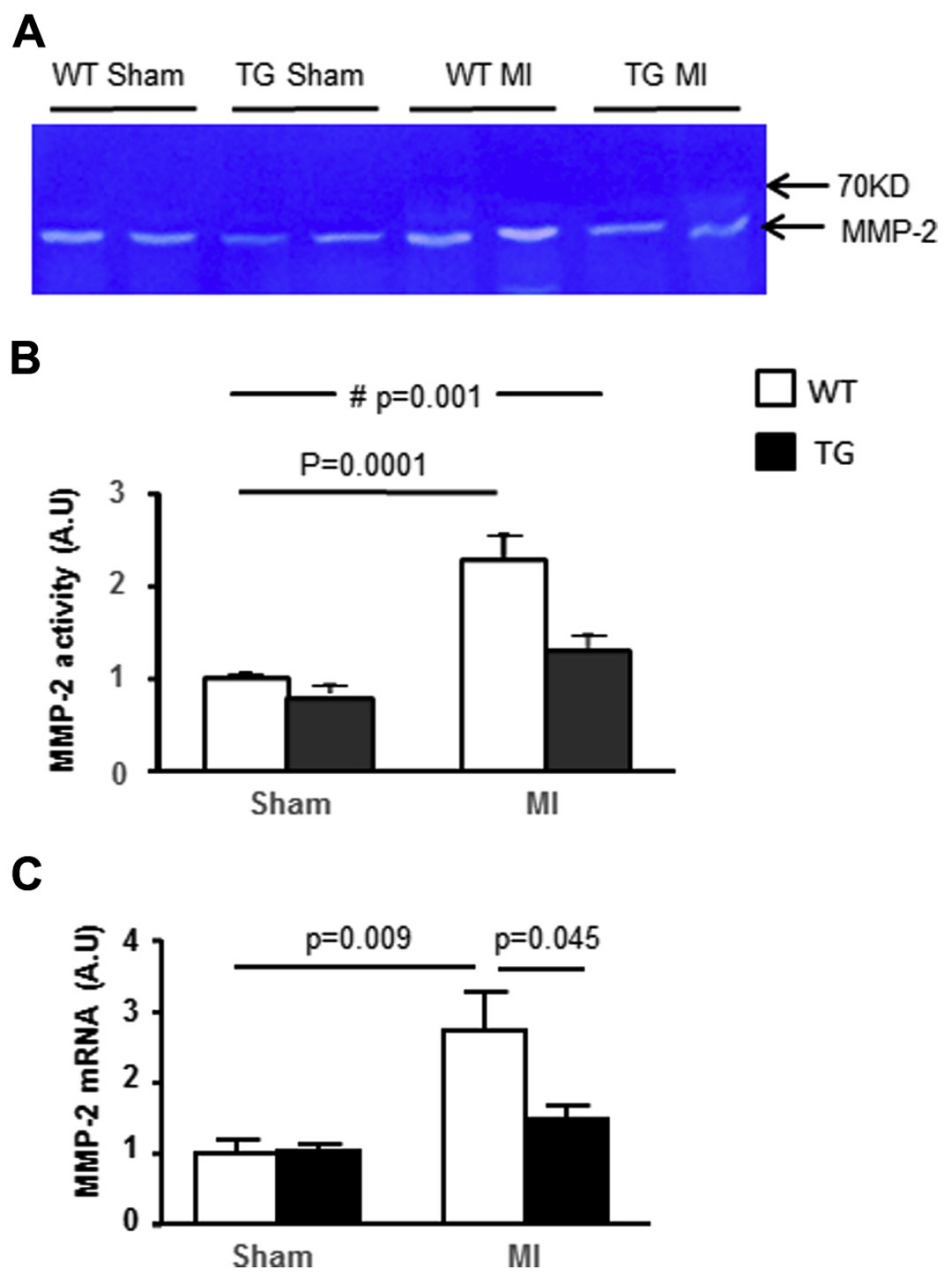
Cardiomyocyte-Targeted Nox4 Alters MMP-2 Activity Profile After I/R Left ventricular matrix metalloproteinase (MMP)-2 activity measured by using gelatin zymography. **(A)** Representative zymogram. **(B)** Mean data. **(C)** mRNA levels of MMP-2 in the left ventricle of WT and TG hearts after ischemia/reperfusion (I/R). n = 5 to 6 per group. #Significant interaction according to 2-way analysis of variance. Abbreviations as in Figures 1, 2, and 5.

To identify mechanisms underlying the higher post-MI survival and reduced adverse remodeling observed in TG mice, we investigated the matrix metalloproteinase (MMP) system. Macrophages secrete different MMPs (notably MMP-2 and MMP-9), which alter the extracellular matrix, hence participating in cardiac tissue remodeling and predilection to cardiac rupture 15, 16, 17. Zymography revealed a significant increase in cleaved MMP-2 after MI in the WT group, but this increase was substantially attenuated in TG myocardium (Figures 6A and 6B). MMP-2 mRNA levels were also increased in WT hearts after MI, a response that was blunted in TG (Figure 6C). MMP-9 was not detected in the cardiac tissue extracts. These results suggest that at least part of the effect of Nox4 on post-MI repair may be linked to changes in MMP activity.

## Discussion

In this study, we found that an elevation of cardiomyocyte Nox4 levels alters the baseline “inflammatory” environment within the myocardium, resulting in a modestly increased number of resident macrophages. More strikingly, cardiomyocyte Nox4 alters macrophage polarization toward an alternatively activated M2 phenotype, especially after myocardial ischemia or I/R, where the changes are evident particularly in the nonischemic myocardium. The change in myocardial macrophage polarization is associated with beneficial effects on post-MI survival and adverse remodeling. These results suggest that an increase in cardiomyocyte Nox4 levels during myocardial ischemia can exert beneficial effects through modulation of macrophage polarization and activation status within the myocardium.

In contrast to other Nox proteins such as Nox1 and Nox2, Nox4 activity depends predominantly on its abundance, and it continuously generates low levels of H_2_O_2_ without requiring acute activation (7). By contrast, Nox2 requires acute activation and generates bursts of superoxide. Diverse stress stimuli, including hypoxia, ischemia, starvation, endoplasmic reticulum stress, neurohumoral stress, and chronic pressure overload, may increase cardiac Nox4 levels 7, 18. In the cardiomyocyte-targeted Nox4 TG mice used in the present study, we reported that myocardial H_2_O_2_ levels are modestly (<2-fold) increased, and there is no evidence of basal cardiac dysfunction or fibrosis at up to 12 months of age (9). Constant low-level H_2_O_2_ generation by Nox4 may promote the transcription of redox-sensitive genes, and here we found an increased expression in TG myocardium of the monocyte chemoattractants CCL2 (i.e., monocyte chemotactic protein–1) and CCL5 (i.e., RANTES), and vascular cell adhesion molecule–1. Levels of the CCL2 receptor, CCR2, were also increased in TG myocardium. These proteins are known to be redox sensitive and involved in monocyte recruitment into tissues, and they may therefore be involved in the observed changes in resident macrophage population 19, 20, 21. Interestingly, previous studies implicated Nox4 in the expression of monocyte chemotactic protein–1 and vascular cell adhesion molecule–1 in endothelial cells (22). Furthermore, recent studies in different mouse models with Nox4 perturbation have suggested a link between Nox4 and inflammatory status (e.g., in atherosclerosis or angiotensin II–induced vascular remodeling) 11, 12, 23. The present study, however, is the first to examine the details of “inflammatory” changes evoked by Nox4 in the heart and to identify changes in macrophage polarization as a major feature.

Although the changes in monocyte chemoattractants may be responsible for the increased number of macrophages in the myocardium, it is conceivable that direct paracrine effects of H_2_O_2_ on monocytes/macrophages could also be involved. Interestingly, a tissue-scale gradient of H_2_O_2_ generated by dual oxidases (one of the Nox family oxidases) contributes to rapid leukocyte influx to the wound site during tail fin regeneration in zebrafish (24). A similar H_2_O_2_-mediated “wound to leukocyte signaling” is also observed in *Xenopus tropicalis* (25). This finding suggests that Nox/H_2_O_2_-dependent effects on inflammatory cell influx may be an evolutionarily conserved wound repair mechanism. H_2_O_2_ could also be involved in the modulation of macrophage polarization observed in the present study. Complex mechanisms regulate the polarization of macrophages into different phenotypes 4, 5. Although skewing toward an M1 phenotype may be driven by lipopolysaccharide and interferon gamma, polarization toward an M2 phenotype requires cytokines (e.g., IL-4, IL-10, IL-13) regulated by nuclear factor–κB (NF-κB) family transcription factors. Previous studies showed that this process may be modulated by H_2_O_2_, potentially via altered NF-κB activation (26). NF-κB–dependent changes in polarization toward an M2 phenotype are also described in tumor-associated macrophages (27).

Macrophage polarization is a key aspect of post-MI wound healing and remodeling. M2 macrophages are recruited between days 4 and 7 after MI in the mouse, later than M1 macrophages, and are suggested to be more anti-inflammatory and reparative 2, 3. M1 and M2 macrophages differ in the cytokines and other factors they secrete. They also differ in their pattern of MMP expression and secretion. M1 macrophages produce high levels of MMPs (e.g., MMP-1, -2, -3, -10) both in vitro and in atherosclerotic plaques, whereas M2 macrophages produce much lower levels (28). We found that MMP-2 expression and zymographic activity were lower in TG hearts than in WT hearts after ischemia, consistent with the inverted M1/M2 balance found in these animals. It should be noted, however, that other cell types (e.g., cardiomyocytes, fibroblasts, endothelial cells) could also contribute to MMP production. MMP activation is known to be linked to post-MI cardiac rupture and also regulates post-MI remodeling 15, 16. Previous studies in mice lacking MMP-2 found that they were protected against post-MI cardiac rupture and adverse remodeling (17). It is therefore likely that the lower MMP-2 activity in the TG myocardium observed in the present study may have contributed to the higher post-MI survival and better post-MI contractile function and remodeling in these animals.

## Conclusions

In this study, we modeled the effects of a rise in cardiomyocyte Nox4 and its effects on the response to MI. The level of Nox4 overexpression was in a pathophysiologically relevant range (9), and our results suggest that cardiomyocyte Nox4 has paracrine effects on macrophages, potentially through altered expression of chemotactic factors as well as via the direct effects of H_2_O_2_. It is conceivable that increases in Nox4 in other cell types (e.g., endothelial cells) could also modulate inflammation, although this theory was not studied. The present study does not exclude the possibility that other Nox4-mediated signaling events previously described in diverse settings (e.g., altered angiogenesis or altered stress responses) (18) may also have contributed to the beneficial effects on post-MI remodeling. However, this study is the first to identify a role for Nox4 in modulating tissue inflammatory profile and macrophage phenotype in the heart, with beneficial effects on the response to both reperfused and nonreperfused MI. A similar modulatory effect of Nox4 in other disease setting (e.g., in atherosclerosis or cancer) could also be pathophysiologically relevant. These results add to an increasing body of evidence that Nox4 exerts complex, often beneficial, effects in the cardiovascular system (18). It should be noted that some studies also reported detrimental effects of increased Nox4, which could be related to overexpression outside a pathophysiologically relevant range. In summary, cardiomyocyte Nox4-driven macrophage polarization protects against post-MI mortality and adverse remodeling.

Perspectives **COMPETENCY IN MEDICAL KNOWLEDGE:** Inflammation is a major determinant of post-MI fate. Different macrophage subsets have different effects, influencing both early survival and later remodeling. Nox family ROS-generating proteins are activated after MI. The Nox4 isoform has potentially beneficial effects by altering macrophage polarization toward a more reparative subtype.

**TRANSLATIONAL OUTLOOK 1:** Approaches to alter macrophage polarization after MI are of interest to study.

**TRANSLATIONAL OUTLOOK 2:** It may be valuable to develop ways of enhancing the beneficial effects of Nox4.
