# Supplementary material for: NADPH Oxidase-4 Driven Cardiac Macrophage Polarization Protects Against Myocardial Infarction–Induced Remodeling
Source: JACC Basic Transl Sci. 2017 Dec 25;2(6):688–98. doi: 10.1016/j.jacbts.2017.06.006 (PMC5803556; doi:10.1016/j.jacbts.2017.06.006)
Supplement: Supplemental Figures 1–7 [file mmc1.pdf]

SUPPLEMENTARY MATERIAL

NADPH oxidase-4 driven cardiac macrophage polarization protects against myocardial infarction-induced remodeling. Heloise Mongue-Din et al

SUPPLEMENTARY FIGURE LEGENDS

Supplementary Figure 1. mRNA levels of SOD1, SOD2 and SOD3 in the hearts of WT and TG mice. n=3-4 per group.

Supplementary Figure 2. Method for flow cytometric characterization and quantification of macrophages in the myocardium.
Single cell suspensions were prepared from the LV or specific LV regions. $CD45^+$ identification was followed by exclusion of doublets and negative identification for CD3/CD19/NK1.1/Ly6G (Dump). Dead cells were excluded based on 7-AAD staining. Macrophages were identified as $(F4/80/CD11b)^+$. Further characterization was based on the expression pattern of Ly6c or MRC1.

Supplementary Figure 3. Charcterization of circulating monocytes in WT and TG mice.
Quantification of the expression of Ly6c and MRC1 in WT and TG animals 3 days after ischemia/reperfusion (I/R), permanent LAD ligation (MI) or a Sham procedure. No differences were observed between genotypes. Results are mean ± SEM; n=5-6 per group.

Supplementary Figure 4. mRNA levels of the cytokines IL-6, IL-10 and TNFα. n=6 per group.

Supplementary Figure 5. Characterization of macrophage polarization in the infarct region and remote region after ischemia-reperfusion (I/R).
A-C) Representative pseudocolor plots for $CD45^+$ expression, Quantification of total leukocytes ($CD45^+$) and macrophages ($F4/80^+$) in the LV of control and TG animals 4 days after MI. D, E) Representative pseudocolor plots for macrophages based on Ly6c or MRC1 expression. Results are mean ± SEM; n=5-6 per group.

Supplementary Figure 6. Representative images of interstitial fibrosis in remote region of WT and TG mice 4 weeks after induction of permanent MI.
Myocardial sections were stained with Picrosirius Red and imaged under normal light (left panel) or polarized light (right panel). Scale bars: 100 µm.

Supplementary Figure 7. Nox2 mRNA levels in WT and TG mouse hearts after permanent MI. n=6 per group.

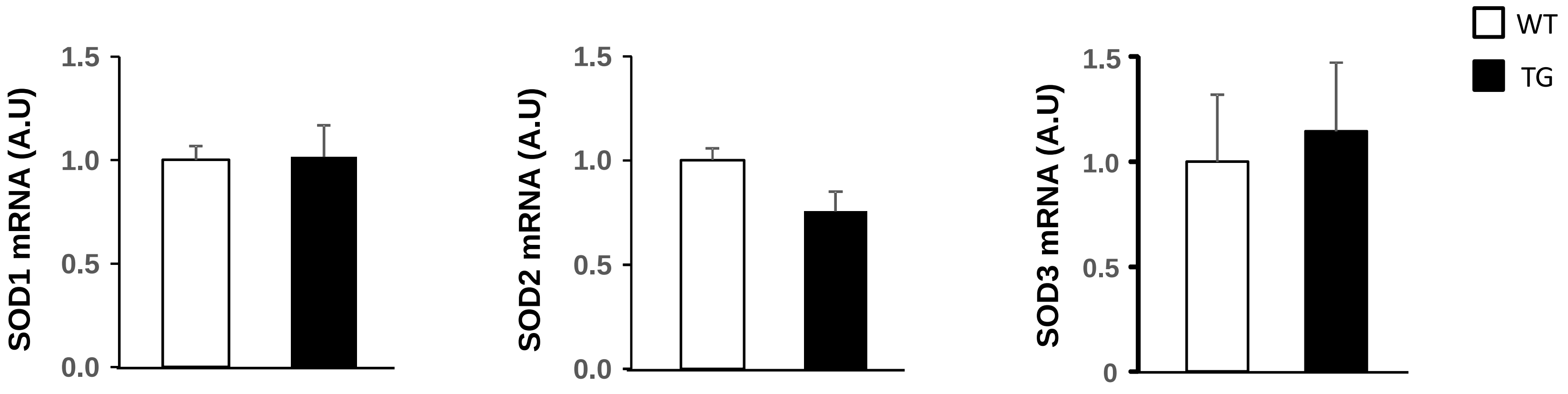

Supplementary Figure 1

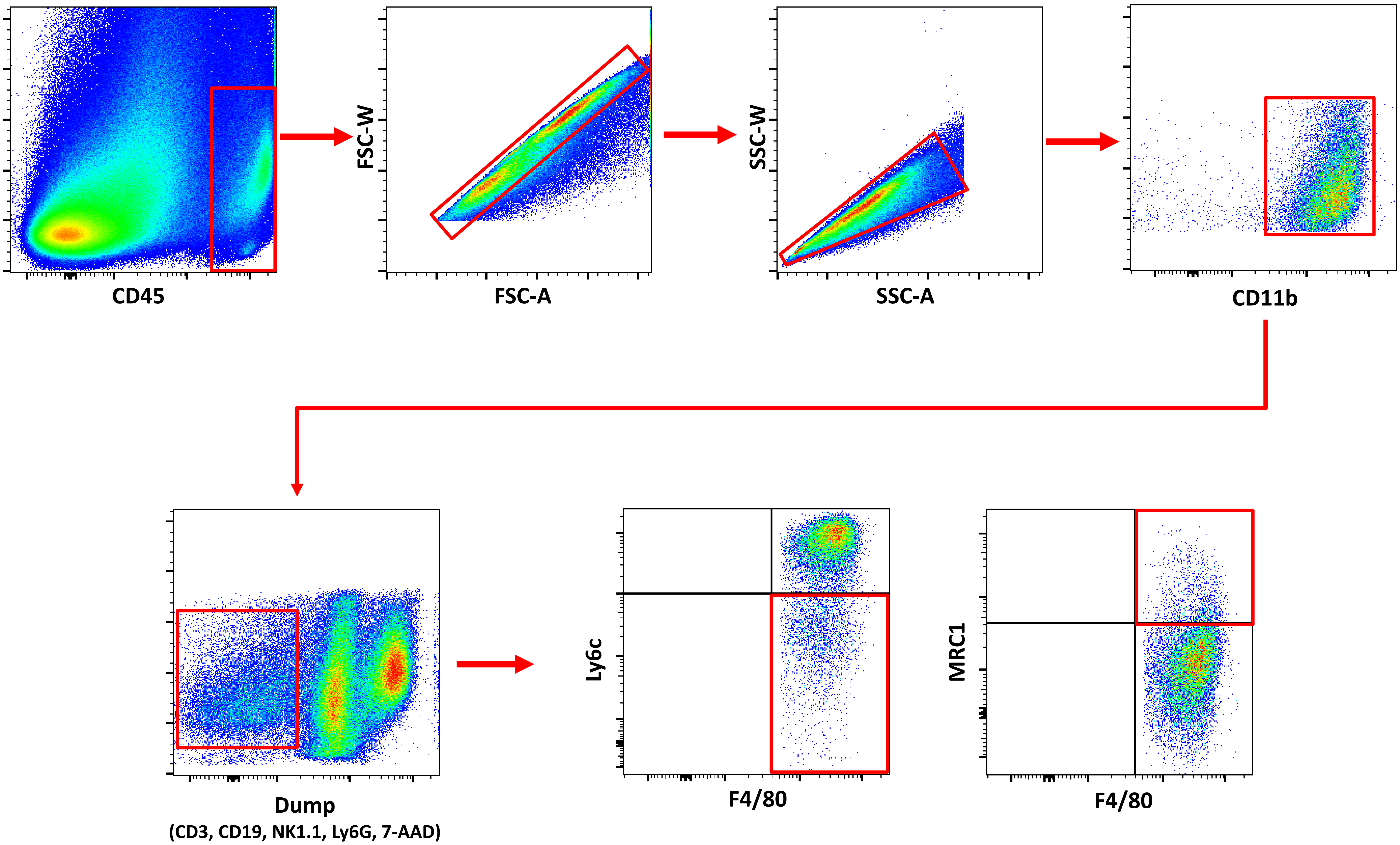

Supplementary Figure 2

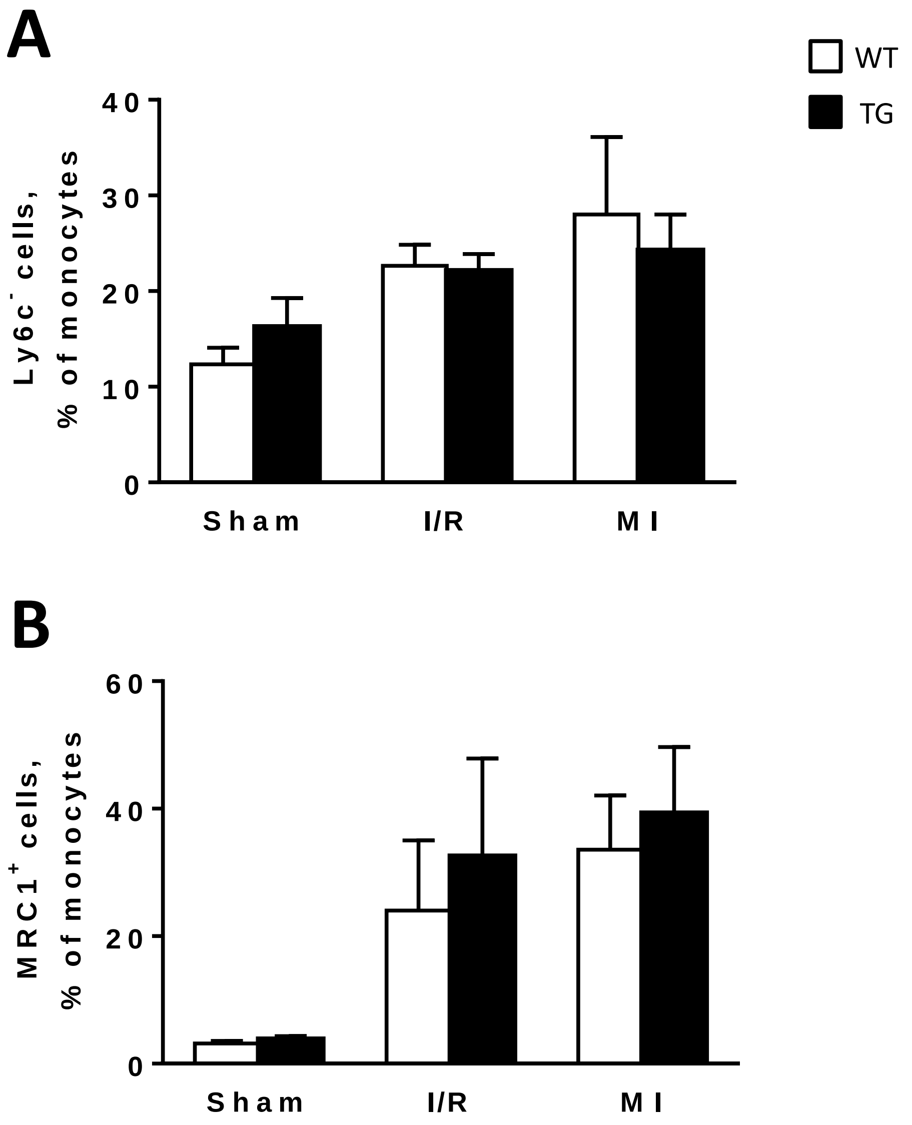

Supplementary Figure 3

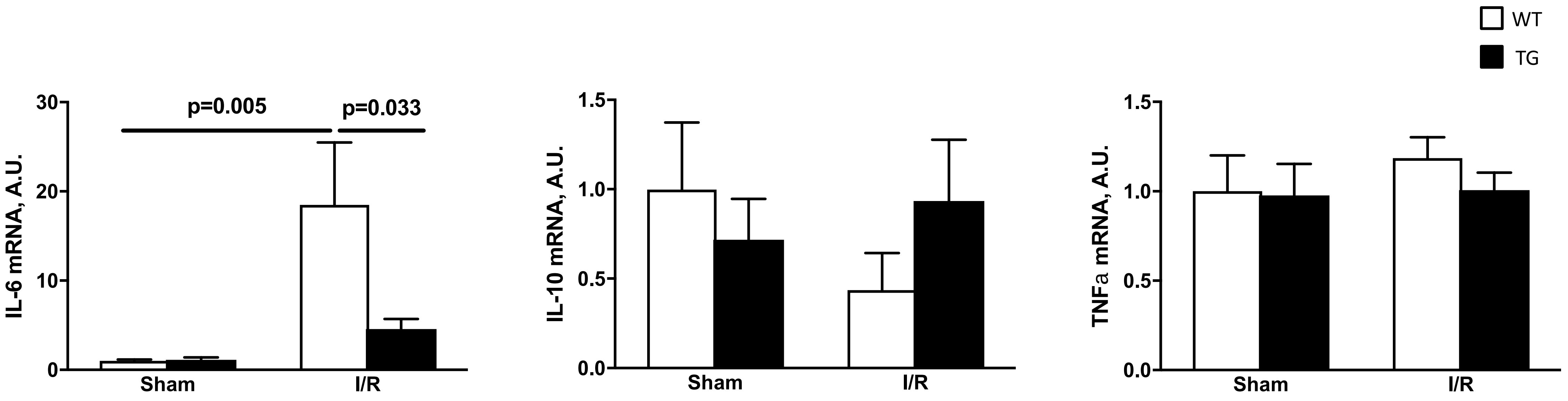

Supplementary Figure 4

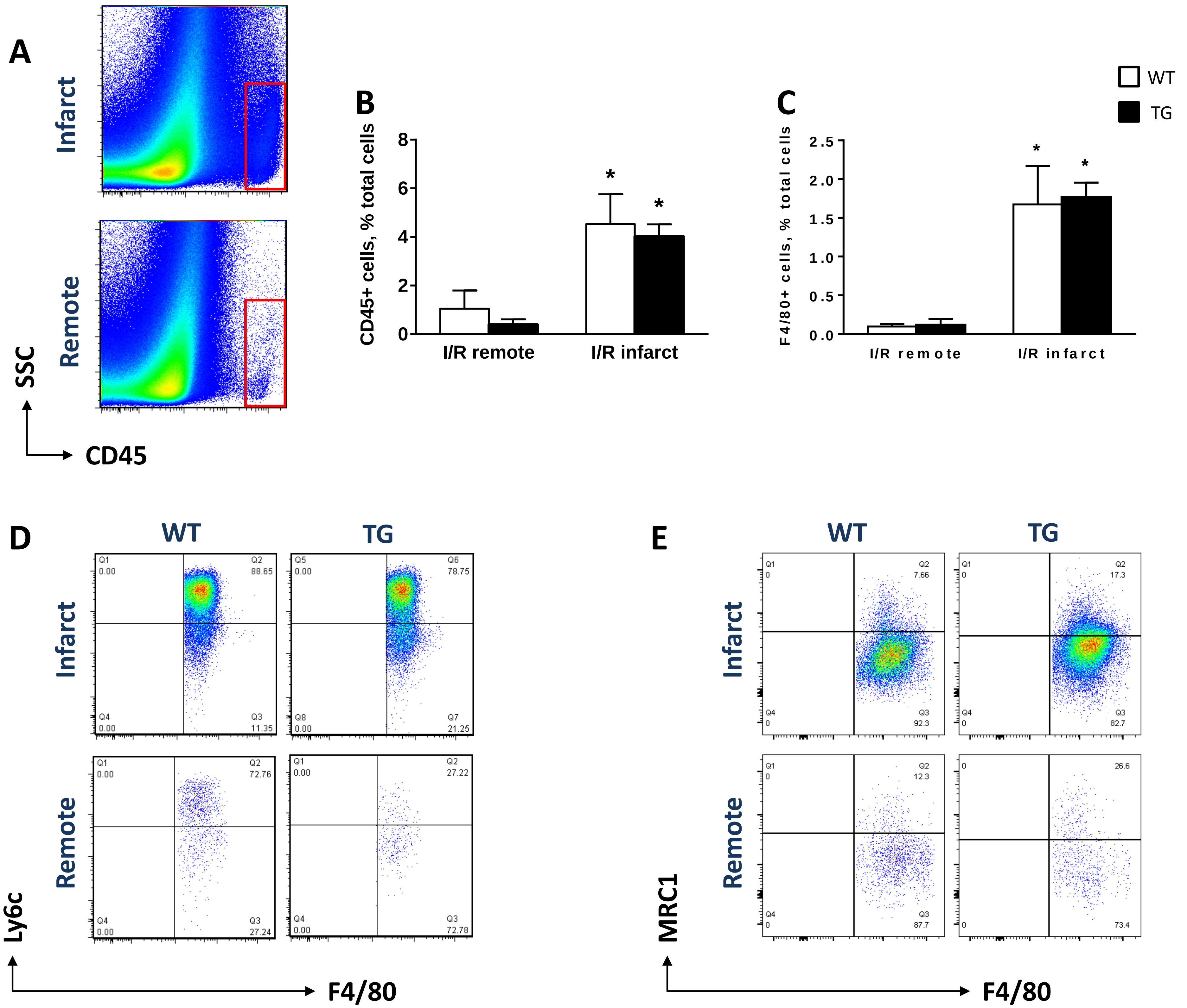

Supplementary Figure 5

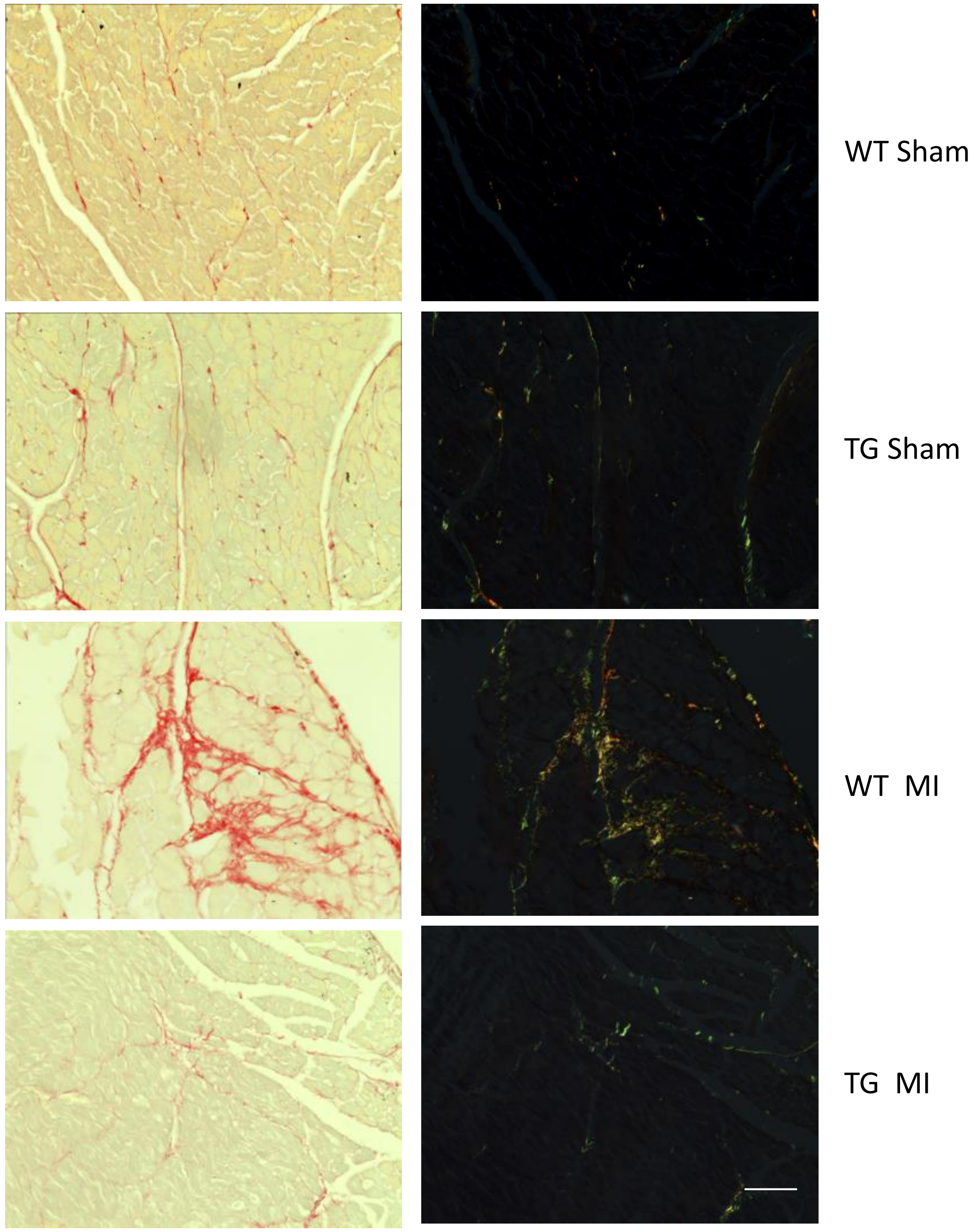

Supplementary Figure 6

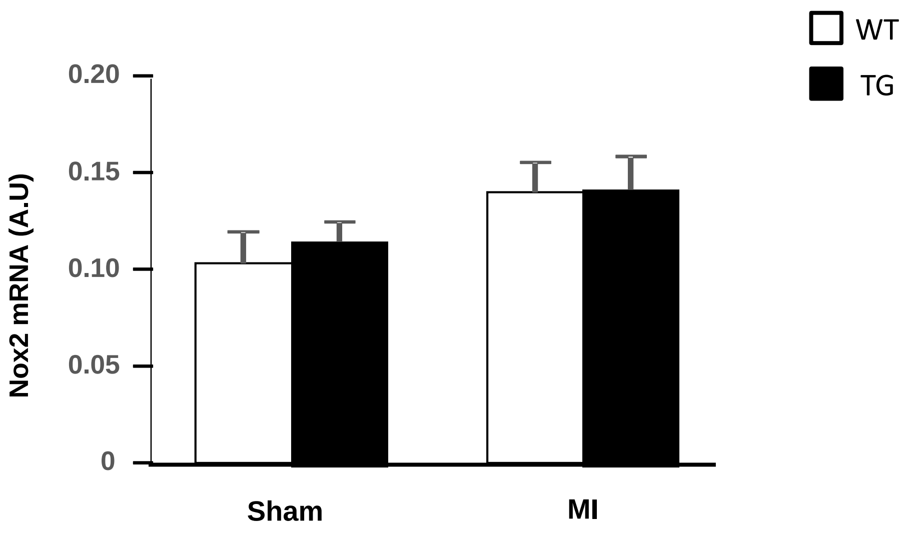

Supplementary Figure 7
